# Telomere Dynamics in Keloids

**Published:** 2011-03-16

**Authors:** Mark Granick, Masayuki Kimura, Soyeon Kim, Lily Daniali, Xiaojian Cao, Utz Herbig, Abraham Aviv

**Affiliations:** ^a^Division of Plastic Surgery, Department of Surgery; ^b^Center of Human Development and Aging; ^c^Department of Preventive Medicine and Community Health; ^d^Department of Microbiology and Molecular Genetics, New Jersey Medical School, University of Medicine and Dentistry of New Jersey, Newark

## Abstract

**Objective:** Little is known about telomere dynamics in keloids. As keloid formation is dependent on cell replication, in theory telomeres should be shorter in keloids than in normal skin. We examined this concept in the present study. **Methods:** We measured by Southern blot analysis telomere length in keloids and in adjacent normal skin of 16 individuals. When available, we also measured telomere length in blood (leukocytes) and subcutaneous fat. **Results:** Telomere length was highly variable among individuals but highly correlated among tissues (cells) within the individual. The mean telomere length in the keloids was longer than that in the adjacent normal skin and displayed a length gradient, with the mean length of telomeres shorter just below the epidermis and longer at the base of the keloids. No apparent telomerase activity was detected in the keloids. **Conclusions:** Our findings suggest a transient activation of telomerase, the reverse transcriptase that prevents telomere shortening, probably during the early phase of keloid formation. The activation of telomerase serves to maintain (or even elongate) telomere length in the keloid. However, telomerase activity is repressed in the fully developed keloid.

## INTRODUCTION

Keloids are benign tumors that result from abnormal wound healing. Persons with dark skin, for example, those of recent African or Asian origins, are more prone to keloids than fair-skinned whites of European ancestry.^1^,^2^ These fibro-proliferative tumors are more common in adolescents and young adults than in older persons and they occur more frequently in the head, neck and trunk than in the lower extremities. Keloids display a high recurrence rate postresection and there are no effective modalities for their prevention (short of avoiding skin injury) in individuals at risk. Various medical treatments to prevent keloid recurrence after resection have been employed with only limited success.^1^,^2^

The histology and composition of keloids have been well-characterized, but their pathogenesis is poorly understood, perhaps because there is no animal model for keloid formation.^3^ Keloid composition includes fibroblasts, inflammatory cells, an abundance of extracellular matrix, primarily collagen, and supportive vascular network. Previous research suggests the potential role of cytokines, principally interleukin 6 (IL-6),^4^ transforming growth factor beta (TGFβ),^5^,^6^ and mesenchymal stem cells^7^ in keloid pathogenesis.

Keloid formation or, for that matter, any wound healing depends on cell proliferation. As somatic cells replicate, their telomeres, the TTAGGG tandem repeats at the ends of chromosomes, undergo progressive shortening.^8^,^9^ Thus, telomere shortening is an index of the replicative history of somatic cells, unless mechanisms that counteract telomere shortening are activated. One of these is the activation of telomerase—a reverse transcriptase that adds telomere repeats to the ends of chromosomes^9^; the other is referred to as the alternative lengthening of telomeres (ALT).^10^ Accordingly, understanding telomere dynamics in keloids, which can become quite large, might shed light on potential mechanisms that sustain their growth over and above that of a normal wound healing. We explored this concept in the present work by measuring telomere lengths in keloids.

## METHODS

### Subjects

We measured telomere length in keloids, in adjacent normal skin, and, when available, in subcutaneous fat and blood (leukocyte) samples from 16 individuals. They included 13 African Americans, 2 Hispanics, and 1 Asian; 8 males and 8 females, with age range from 14 to 58 years.

### Keloid resection and sampling of subcutaneous fat and blood

Most keloids were resected under local anesthesia. The lesion was removed with minimal injury to adjacent normal skin. In some cases, keloids were removed under general anesthesia. In that setting, the anesthesiologist obtains a blood sample while inserting an intravenous catheter. Subcutaneous fat tissue was obtained only when fat was exposed in the operative field.

### Measurements of telomere length and telomerase activity

DNA was extracted by phenol/chloroform and telomere length was measured by Southern blot analysis of the terminal restriction fragments (TRFs).^11^ All DNA samples were subjected to integrity testing; no sample displayed evidence of DNA degradation. Telomerase activity was measured by the TRAP assay (TRAPeze Telomerase Detection kit, Millipore, California). In 9 keloids, we mapped telomere length in 3 distinct regions: the most distant area from the surgical excision, just below the epidermis (keloid 1); area adjacent to the normal skin at the base of the keloid (keloid 2); and the center of the base of the keloid (keloid 3). The remaining 7 keloids were small and the entire keloid was used to measure telomere length.

As noted earlier, we obtained keloid and skin samples from the 16 participants but could not always obtain a complete set of samples (including keloid, skin, subcutaneous fat, and blood).

### Statistical analysis

We used the mixed procedure in SAS 9.1 to perform repeated-measures ANOVA. Results from all participants were used for analyses, unless otherwise indicated. We assumed unstructured correlations between measures within the same individual to compare telomere lengths in different tissues or keloid regions. The model comparing skin and keloids used the following categories: keloid (in the case that the entire keloid was used), keloid 1, keloid 2, or keloid 3 as defined earlier. Least square means and confidence intervals (CIs) from the model are presented. A contrast was used to compare the mean telomere length in skin to keloids; since we had a single keloid sample for some patients but up to 3 samples from different regions of the tumor for others, we performed a 2-*df* test contrasting the mean telomere length in skin to that in the single keloid sample and the mean telomere length in skin to the average of means from the 3 keloid regions. A subset analysis of those who provided samples from 3 keloid regions was also conducted to confirm results. We also performed a less conservative test comparing skin and keloid by fitting a model with an indicator of whether a sample came from skin or keloid, assuming a compound symmetry correlation structure, and then compared telomere length in skin to keloids using a 1-*df* test. Pairwise comparisons were conducted to identify differing categories.

All testing was 2-sided and conducted at the 5% significance level.

### Informed consent

All participants provided written informed consents approved by the institutional review board of the University of Medicine and Dentistry of New Jersey, New Jersey Medical School.

## RESULTS

**Figure 1:**
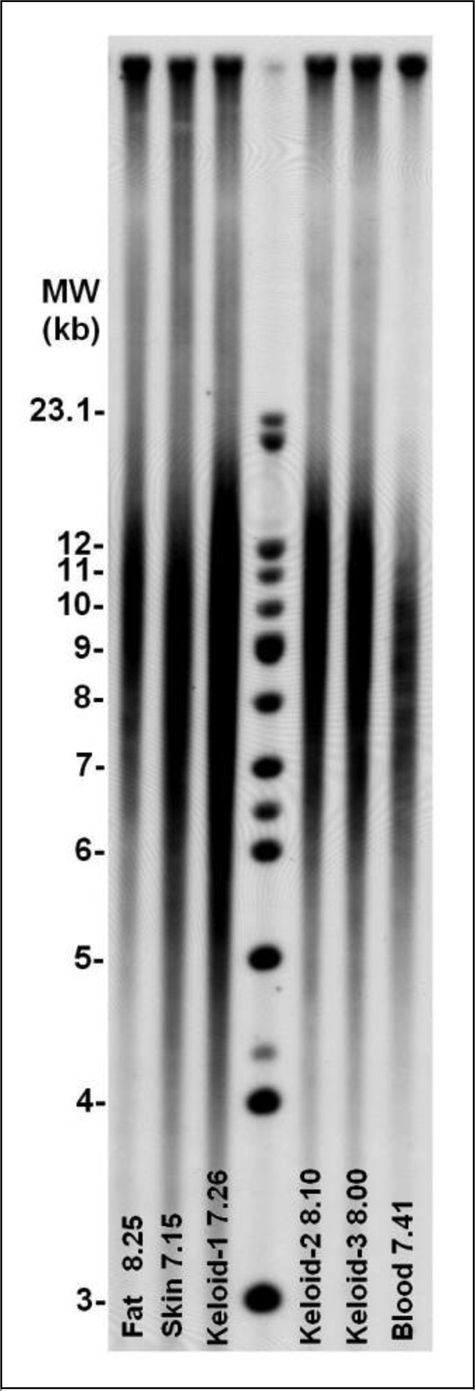
Southern blots of the terminal restriction fragments. The source of the DNA and the mean length (in kb) of the terminal restriction fragments are indicated at the bottom of each lane. The molecular-weight reference ladder is shown in the fourth lane from the left.

Figure 1 illustrates typical Southern blots analysis of the TRFs from subcutaneous fat, normal skin, 3 regions in the keloid (keloid 1, keloid 2, and keloid 3) and blood samples of a single individual.

**Figure 2:**
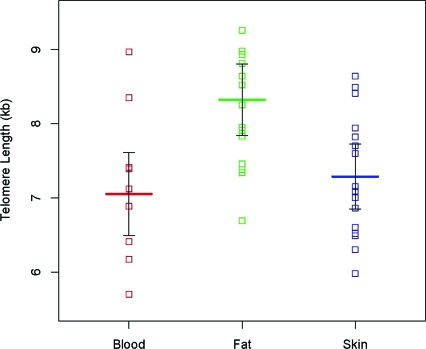
Telomere length in subcutaneous blood, fat, and skin in patients with keloids. Broad horizontal lines are the mean telomere lengths and vertical bars are 95% confidence intervals based on repeated-measures analysis. Omnibus test of any difference by tissue type: *P* < .001. Fat is significantly different from both blood and skin (both *P* < .001), but blood and skin are not significantly different from each other (*P* = .27).

Figure 2 shows telomere length in subcutaneous fat, normal skin, and blood from the 16 study participants. The mean telomere length in subcutaneous fat, 8.32 kb (95% CI: 7.84–8.81) was longer than that in skin, 7.29 kb (95% CI: 6.85–7.73), and blood, 7.05 kb (95% CI: 6.49–7.61), and the differences were statistically significant (*P* < .001), but there was no difference in mean telomere length between skin and blood (*P* = .27). Models that further adjusted for age and whether the participant was African American did not qualitatively change the estimated telomere lengths or conclusions comparing lengths in skin and keloids. Younger age (*P* = .077) and African Americans (*P* = .090) had marginally significant longer mean telomere lengths in the 3 tissues. We could not formally test for differences in telomere lengths in tissues by age and being African American because of the small sample size.

**Figure 3:**
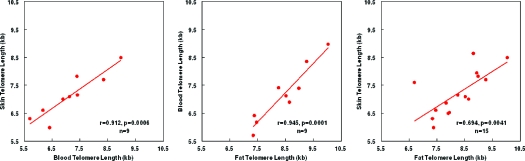
Synchrony in telomere length. Skin vs blood (left panel); blood vs subcutaneous fat (middle panel); and skin vs subcutaneous fat (right panel). Pearson correlations are shown, *P* value test whether the correlation = 0.

Figure 3 displays the relation of telomere lengths between normal skin and blood, between normal skin and subcutaneous fat, and between blood and subcutaneous fat. The interindividual variation in telomere length among participants amounted to approximately 3 to 3.5 kb. However, relative synchrony in telomere length was found for these tissues within the individual so that a person who had long (or short) telomeres in one tissue tended to have long (or short) telomeres in other tissues. Pairwise scatterplots of telomere lengths did not always fall along the 45° line, which would suggest equal lengths in tissues; specifically, comparisons of lengths in fat to the other tissues show that telomeres in fat are generally longer than lengths in skin or blood, consistent with the results from the repeated-measures ANOVA.

**Figure 4:**
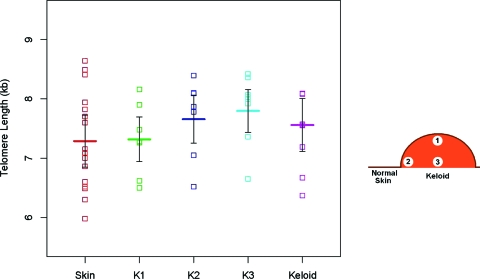
Telomere length in normal skin and in keloid samples. Normal skin, different regions in keloids when available; keloid 1 (K-1), keloid 2 (K-2), and keloid 3 (K-3) or the entire keloid, when the entire keloid was used to determine telomere length. Broad horizontal lines are the mean telomere lengths and vertical bars are 95% confidence intervals based on repeated-measures analysis. Omnibus test comparing the mean telomere lengths in the 5 categories: *P* = .029. Two-degree-of-freedom test comparing mean telomere length in skin vs keloid and in skin vs average of K-1, K-2, and K-3: *P* = .14. Pairwise comparisons of mean telomere lengths in skin vs K-1: *P* = .84, skin vs K-2: *P* = .11, skin vs K-3: *P* = .012, skin vs keloid: *P* = .14, K-1 vs K-2: *P* = .023, K-1 vs K-3: *P* = .003, and K-2 vs K-3: *P* = .25.

Figure 4 shows the relation between telomere lengths in normal skin specimens and in keloids. The least square means (95% CI) for telomere length in normal skin was 7.29 kb (6.85–7.73), at keloid 1 was 7.32 kb (6.94–7.69), at keloid 2 was 7.66 kb (7.25–8.06), and at keloid 3 was 7.80 kb (7.43–8.16) and for those in which telomere length was measured using the entire keloid was 7.56 kb (7.11–8.00). The overall test was significant (*P* = .029), suggesting a difference within the keloid regions or between the normal skin and one of the keloid regions. There was no evidence of telomere shortening in the keloid compared with normal skin. In fact, there was a tendency for a longer telomere length in keloid tissue than in the adjacent normal skin. Pairwise comparisons suggest that the mean telomere length at keloid 3, sampled from the base of the keloid, was longer than both adjacent normal skin and keloid 1. Keloid 1 and keloid 2 were also significantly different. Telomere length showed the following pattern: normal skin < keloid 1 < keloid 2 < keloid 3. A subgroup analysis of those patients with results from skin and each of the three keloid regions showed a similar pattern.

Although the 2-*df* test comparing mean telomere length in skin versus keloid and in skin versus average of keloid 1, keloid 2, and keloid 3 was not significant (*P* = .14), a simpler model with a covariate for skin versus keloid, treating the keloid measurements as indistinguishable, allowed us to do a more powerful 1-*df* comparison of skin versus keloids, which gave *P* = .041. Neither keloid tissue nor normal skin showed detectable telomerase activity.

## DISCUSSION

The runaway growth that forms a keloid is poorly understood, but the present study indicates that it might be linked to telomere dynamics in the growing keloid. In spite of the proliferative activity necessary to sustain keloid growth, telomere length in the keloid mass is not shorter than that in the adjacent normal skin.

Figure 2 underscores the impact of somatic cell proliferation on telomere length, as it shows that telomere length is considerably longer in subcutaneous fat than telomere length in skin and leukocytes. While subcutaneous fat is poorly proliferative, skin and the hematopoietic system are highly proliferative. Yet within the individual, telomere length is highly correlated among different tissues, a finding also observed in previous work in humans and macaques.^12^^-^15 We attribute this phenomenon to the lasting effect of birth telomere length, which is highly synchronized among tissues within the newborn but is highly variable across newborns.^15^ Thus, individuals with long telomeres in one tissue are likely to have long telomeres in other tissues. That said, Figure 3 suggests that the predilection to keloid formation does not relate to the individual's telomere length, since the study participants exhibited a wide range of telomere length that overlaps that shown in other studies of individuals not selected on the basis of having keloids.

A previous study observed no telomerase activity in keloids but concluded that keloid telomere length was shorter than that in normal skin.^16^ We have confirmed the lack of telomerase activity in the keloid. However, our data indicate that telomere length in keloids is not shorter than that of normal skin. In the previous study,^16^ keloids were obtained from one group of individuals, while normal skin specimens were obtained from another group. Given the large interindividual variation in age-adjusted telomere length, such a design requires a large number of participants and that controls be carefully selected to avoid bias. Our design has controlled for the high interindividual variation in telomere length by comparing telomere length in the keloid with that in adjacent normal skin of the same individual. In addition, the previous work showed in a subset of samples a strong TRF signal that extended to the very low-molecular-weight region (Figure 2 in De Felice et al^16^, which suggests compromised DNA integrity. We also note the inadequate resolution of the Southern blots in that study, shown in the illustrative Figure 2, and presume that the molecular weight ladder in the figure may have been misplaced, as the mean TRF of normal skin specimens appeared to be more than 1 kb shorter than the ref 6.1 molecular weight marker.

Keloids can attain a large mass, which entails considerable proliferation of their cellular elements. They are usually excised at a later phase of their growth after attaining a size that causes disfigurement. Therefore, the inevitable conclusion of findings that telomere length in keloids is not shorter and in fact might be longer than in normal skin is that mechanisms that prevent telomere shortening operate during keloid formation. Telomerase^8^ and on rare occasions ALT^10^ are such mechanisms. It is unlikely that activation of the ALT pathway explains the lack of telomere shortening in keloids, because this mechanism primarily operates in various forms of cancer and immortalized cell lines, which often display aberrant chromosomal configurations, genomic instability, and relatively short telomeres, which is not the case for keloids. Given that we detected no apparent telomerase activity in mature keloids, we hypothesize that telomerase activation is transient and primarily takes place during the early growth phase of keloids; by the time the keloid is excised, telomerase activity is apparently repressed.

To underscore the difference between telomere dynamics in keloids versus cancer, it is useful to compare the clonal evolution model of cancer (reviewed in Visvader and Lindeman^17^) and the model we propose for keloids. The clonal evolution model posits that mutated but preneoplastic cells with growth advantage undergo multiple divisions, which cause progressive telomere attrition until critically shortened telomeres set the limit for further replication. On a rare occasion, telomerase, or less frequently ALT, is activated as the cells undergo complete neoplastic transformation. However, telomerase activation rarely serves to elongate telomeres when cells undergo cancerous transformation; it just prevents further shortening. The clonal evolution model thus predicts that cancer would often display shorter telomeres than adjacent noncancerous tissue in concert with robust telomerase activity.^18^^-^20

Our model for telomere dynamics in keloid presumes that telomerase is activated only during the early phase of the abnormal wound healing that brings about the formation of keloids, and perhaps other hypertrophic scars. In contrast to the scenario in cancer where the activation of telomerase prevents further telomere shortening, telomerase activation in normal (precursor) cells of the forming keloid not only prevents the shortening of telomeres but also might elongate them. As the keloid mass increases in size, the stimulation of telomerase gradually declines, such that by the time of the resection of the keloid, telomerase activity is ostensibly undetectable. In this context, our finding that telomere length is shorter just under the epidermis than at the base of the keloid suggests that the formation of the keloid proceeds from a growth zone under the epidermis toward the base. In this way, cells at the base of the keloid are those formed shortly after the healing process has started, when telomerase activity is presumably at its peak and their telomeres are, therefore, the longest. In contrast, cells toward the surface of the keloid have been formed later in the phase of wound healing when telomerase activity is progressively repressed and consequently their telomeres are relatively shorter. In that light, it is noteworthy that keloid precursor cells were recently isolated from the dermal layer of keloids and these cells display features of stem cells, including telomerase activity.^21^ It would be of interest whether these cells are more abundant at the epidermis/dermis boundary.

Compared to normal wounds or skin, keloids are highly enriched in growth factors, extracellular matrix, and inflammatory cytokines such as IL-6 and TGFβ. And this altered extracellular milieu might play a central role in keloid development.^22^ Some of these cytokines have been implicated in regulating the activity of telomerase. For instance, IL-6 increases telomerase expression and activity in dermal progenitor cells, which is greater in keloid-derived progenitor cells, compared with those derived from normal skin.^21^ High IL-6 levels in keloid scars, combined with increased sensitivity to IL-6-induced telomerase activity, could result in telomere elongation, which would explain why cells in keloids have longer telomeres than cells in normal tissue.

In addition, keloid fibroblasts might have an altered sensitivity to signaling by TGFβ,^6^ which induces trans-differentiation of fibroblast to myofibroblast.^23^ TGFβ is also a negative regulator of the expression of the catalytic subunit of telomerase hTERT^24^^-^27 and it is plausible, therefore, that TGFβ-mediated hTERT repression progressively increases as the keloid matures. Interestingly, in itself, inhibition of telomerase can induce trans-differentiation of fibroblasts into myofibroblasts,^28^ which suggests that TGFβ-induced myofibroblast differentiation might be mediated by hTERT inhibition. However, given that myofibroblasts are absent in keloids,^23^ despite the presence of high levels of TGFβ and its receptors, cells in keloids might be less sensitive to TGFβ-induced telomerase inhibition and trans-differentiation. This would explain the higher abundance of progenitor cells, longer telomeres, and absence of myofibroblasts in keloids.

In conclusion, precancerous cells that ultimately become cancerous already display short telomeres before telomerase activation, which serves to prevent further telomere erosion upon malignant transformation. We propose that in contrast, the abnormal wound healing process that promotes keloid formation starts with normal cells, perhaps mesenchymal stem cells, in which telomerase “hyperactivation” not only prevents telomere shortening due to replication but might even elongate telomeres. This phenomenon would sustain the growth of the keloid at the early phase of its formation, but telomerase activity is repressed once the keloid is well-formed.

On the basis of these considerations, we propose that telomerase activation might be targeted in a deliberate effort to prevent or attenuate keloid growth through topical and intraincisional application of antitelomerase compounds. Telomere length in normal skin of young adults is generally long enough to enable sufficient number of replication before critically shortened telomere length might compromise replication. That said, the topical and intraincisional application of antitelomerase compounds should be closely monitored to ascertain that it does not impair wound healing.

## References

[1] Chike-Obi CJ, Cole PD, Brissett AE (2009). Keloids: pathogenesis, clinical features, and management. Semin Plast Surg.

[2] Mustoe TA, Cooter RD, Gold MH (2002). International clinical recommendations on scar management. Plast Reconstr Surg.

[3] Köse O, Waseem A (2008). Keloids and hypertrophic scars: are they two different sides of the same coin?. Dermatol Surg.

[4] Ghazizadeh M, Tosa M, Shimizu H, Hyakusoku H, Kawanami O (2007). Functional implications of the IL-6 signaling pathway in keloid pathogenesis. J Invest Dermatol.

[5] Chin GS, Liu W, Peled Z (2001). Differential expression of transforming growth factor-beta receptors I and II and activation of Smad 3 in keloid fibroblasts. Plast Reconstr Surg.

[6] He S, Liu X, Yang Y (2010). Mechanisms of transforming growth factor beta(1)/Smad signalling mediated by mitogen-activated protein kinase pathways in keloid fibroblasts. Br J Dermatol.

[7] Akino K, Akita S, Yakabe A (2008). Human mesenchymal stem cells may be involved in keloid pathogenesis. Int J Dermatol.

[8] Blackburn EH (2005). Telomeres and telomerase: their mechanisms of action and the effects of altering their functions. FEBS Lett.

[9] Wong JM, Collins K (2003). Telomere maintenance and disease. Lancet.

[10] Cesare AJ, Reddel RR (2010). Alternative lengthening of telomeres: models, mechanisms and implications. Nat Rev Genet.

[11] Kimura M, Stone RC, Hunt SC (2010). Measurement of telomere length by the Southern blot analysis of terminal restriction fragment lengths. Nat Protoc.

[12] Gardner JP, Kimura M, Chai W (2007). Telomere dynamics in macaques and humans. J Gerontol A Biol Sci Med Sci.

[13] Lukens JN, Van Deerlin V, Clark CM, Xie SX, Johnson FB (2009). Comparisons of telomere lengths in peripheral blood and cerebellum in Alzheimer's disease. Alzheimers Dement.

[14] von Zglinicki T, Serra V, Lorenz M (2000). Short telomeres in patients with vascular dementia: an indicator of low antioxidative capacity and a possible risk factor?. Lab Invest.

[15] Okuda K, Bardeguez A, Gardner JP (2002). Telomere length in the newborn. Pediatr Res.

[16] De Felice B, Wilson RR, Nacca M (2009). Telomere shortening may be associated with human keloids. BMC Med Genet.

[17] Visvader JE, Lindeman GJ (2008). Cancer stem cells in solid tumours: accumulating evidence and unresolved questions. Nat Rev Cancer.

[18] Meeker AK, Hicks JL, Gabrielso E, Strauss WM, De Marzo AM, Argan P (2004). Telomere shortening occurs in subsets of normal breast epithelium as well as in situ and invasive carcinoma. Am J Pathol.

[19] van Heek NT, Meeker AK, Kern SE (2002). Telomere shortening is nearly universal in pancreatic intraepithelial neoplasia. Am J Pathol.

[20] Joshua AM, Vukovic B, Braude I (2007). Telomere attrition in isolated high-grade prostatic intraepithelial neoplasia and surrounding stroma is predictive of prostate cancer. Neoplasia.

[21] Zhang Q, Yamaza T, Kelly AP (2009). Tumor-like stem cells derived from human keloid are governed by the inflammatory niche driven by IL-17/IL-6 axis. PLoS One.

[22] Shih B, Garside E, McGrouther DA, Bayat A (2010). Molecular dissection of abnormal wound healing processes resulting in keloid disease. Wound Repair Regen.

[23] Bran GM, Goessler UR, Hormann K, Riedel F, Sadick H (2009). Keloids: current concepts of pathogenesis. Int J Mol Med.

[24] Cassar L, Li H, Jiang FX, Liu JP (2010). TGF-beta induces telomerase-dependent pancreatic tumor cell cycle arrest. Mol Cell Endocrinol.

[25] Hu B, Tack DC, Liu T, Wu Z, Ullenbruch MR, Phan SH (2006). Role of Smad3 in the regulation of rat telomerase reverse transcriptase by TGFbeta. Oncogene.

[26] Lacerte A, Korah J, Roy M, Yang XJ, Lemay S, Lebrun JJ (2008). Transforming growth factor-beta inhibits telomerase through SMAD3 and E2F transcription factors. Cell Signal.

[27] Li H, Xu D, Li J, Berndt MC, Liu JP (2006). Transforming growth factor beta suppresses human telomerase reverse transcriptase (hTERT) by Smad3 interactions with *c-Myc* and the *hTERT* gene. J Biol Chem.

[28] Liu T, Hu B, Chung MJ, Ullenbruch M, Jin H, Phan SH (2006). Telomerase regulation of myofibroblast differentiation. Am J Respir Cell Mol Biol.

